# A New Polymer-Based Fluorescent Chemosensor Incorporating Propane-1,3-Dione and 2,5-Diethynylbenzene Moieties for Detection of Copper(II) and Iron(III)

**DOI:** 10.3390/polym9070267

**Published:** 2017-07-06

**Authors:** Dongliang Yang, Chunhui Dai, Yanling Hu, Shuli Liu, Lixing Weng, Zhimin Luo, Yixiang Cheng, Lianhui Wang

**Affiliations:** 1 Key Laboratory for Organic Electronics & Information Displays (KLOEID) and Institute of Advanced Materials (IAM), Nanjing University of Posts & Telecommunications, 9 Wenyuan Road, Nanjing 210046, China; 1011071735@njupt.edu.cn (D.Y.); huyanling124@hotmail.com (Y.H.); liushuli_1990@126.com (S.L.); iamzmluo@njupt.edu.cn (Z.L.); 2 Key Laboratory of Mesoscopic Chemistry of MOE, School of Chemistry and Chemical Engineering, Nanjing University, Nanjing 210093, China; daichunhui@163.com; 3 College of Geography and Biological Information, Nanjing University of Posts and Telecommunications, Nanjing 210046, China; lxweng@njupt.edu.cn

**Keywords:** conjugated polymers, sensor, ion detection, copper(II), iron(III)

## Abstract

A novel conjugated polymer (PDBDBM) was developed by the polymerization of 1,4-dioctyloxy-2,5-diethynylbenzene with 1,3-bis(4-bromophenyl)propane-1,3-dione based on Pd-catalyzed Sonogashira-coupling reaction. The obtained polymer PDBDBM exhibited bright green photoluminescence under UV irradiation. According to the metal ion titration experiments, PDBDBM showed high sensitivity and selectivity for detection of Cu^2+^ and Fe^3+^ over other metal ions. The fluorescent detection limits of PDBDBM were calculated to be 5 nM for Cu^2+^ and 0.4 μM for Fe^3+^ and the Stern–Volmer quenching constant for Cu^2+^ and Fe^3+^ were found to be 1.28 × 10^8^ M^−1^ and 2.40 × 10^4^ M^−1^, respectively. These results indicated that the polymer can be used as a potential probe for Cu^2+^ and Fe^3+^ detection.

## 1. Introduction

All organisms have absolute requirements for a series of metals as they play critical role in biological processes [1]. Copper, an essential trace metal, participates in a variety of fundamental physiological processes, such as redox processes and enzyme-catalyzed reactions [2]. Dysregulation of Cu^2+^ homeostasis could lead to numerous diseases, including amyotrophic lateral sclerosis, Parkinson’s disease, Alzheimer’s disease, Menkes disease, and neurodegenerative diseases [2,3]. Iron, as the second most abundant trace metal element, is involved in many physiological events such as oxygen transport, DNA synthesis, and electron transport [3,4]. Iron deficiency is associated with disorders such as anemia, hemochromatosis, and Alzheimer’s [5]. Moreover, Cu^2+^ and Fe^3+^ are ecological pollutions since they are widely used in agriculture and industry [6,7]. Consequently, numerous efforts have been made to develop selective and sensitive detection methods for Cu^2+^ and Fe^3+^, including UV–Vis [8], fluorescence [4], atomic absorption spectra [9], and electrochemical methods [10]. Of these methods, fluorescent probes have been widely used owing to their simplicity, low cost, fast response time, and high sensitivity. Currently, various fluorescent probes for Cu^2+^ and Fe^3+^ have been developed; but most of them are based on small organic molecules, and polymer-based chemosensors are very few [3].

Over the past decades, conjugated polymers (CPs) have gained increasing attention owing to their extensively π-conjugated backbones and conformationally restricted polymer chains [11]. Furthermore, the light-harvesting and light-amplifying properties of CPs make them attractive for applications in optoelectronics, fluorescence imaging, and chemosensors [11,12]. Compared to small organic fluorescent molecule sensors, polymer-based fluorescent chemosensors have several noteworthy features. For example, polymer-based fluorescent chemosensors can enhance and amplify the fluorescence responsive signal which was ascribed to facile energy migration along the polymer backbone upon light excitations [13,14]. Furthermore, polymer-based sensors possess high selectivity and binding efficiency due to the polymer chain incorporating multiple recognition elements for analytes [15]. These CPs show superior performance and offer a new platform to fluorescent sensors [16,17]. Poly(phenylene-ethynylene) (PPE) CPs are expected to possess strong fluorescence, making them amenable for applications such as fluorescent sensor emitters [18]. Many fluorescent sensors based on PPE derivative have been exploited for the detection of small molecule and biomolecule with high selectivity and sensitivity [18]. To develop the selective fluorescent sensors, various recognition moieties such as binaphthalene, oligo-crown ethers, and acetophenone ligands have been introduced to the backbone and side-chain of conjugated polymers [15,19]. Currently, some fluorescent sensors based on CPs for Cu^2+^ or Fe^3+^ have been developed. For example, the fluorescent polymers containing imidazole, bipyridine, and thiourea coordination moieties can serve as fluorescent sensors for Cu^2+^ [8,20,21,22]. Besides, some CPs bearing multidentate amino or carboxylate side chains also display high selectivity toward Cu^2+^ [23,24,25]. For design and synthesis Fe^3+^ sensors, the approach that introducing quinoline, phosphonate sodium salt, sulphonate, spirolactam, terpyridine, and carboxylic ester groups to conjugated polymer chain have been developed [26,27,28,29,30,31]. However, few of them can be used in both Cu^2+^ and Fe^3+^ detection. Moreover, there is no literature about polymer-based fluorescence containing propane-1,3-dione derivatives for selective metal ion detection. In the organic phosphorescent metal complexes, however, propane-1,3-dione derivatives can be used as bridging coordination moiety of metal [32,33].

In the present work, a novel fluorescence-based polymer (PDBDBM) sensor containing 1,4-dioctyloxy-2,5-diethynylbenzene (M1) and 1,3-bis(4-bromophenyl)propane-1,3-dione (M2) was synthesized based on Pd-catalyzed Sonogashira-coupling reaction, which demonstrated obvious absorbance change and fluorescence quenching upon the addition of Cu^2+^ and Fe^3+^ ion without interference from other metal ions. It is worth mentioning that the intensity change of photoluminescence of PDBDBM sensor could be easily observed by the naked eye under a hand-held UV lamp, which proved that PDBDBM can be utilized as potential optical chemosensors for Cu^2+^ and Fe^3+^ detection.

## 2. Materials and Methods

### 2.1. Materials and Measurements

All of the reagents and solvents were commercially available and of analytical reagent grade. Triethylamine (Et_3_N) and tetrahydrofuran (THF) were purified by distillation as previously described [34]. 1,4-dioctyloxy-2,5-diethynylbenzene (M1) and 1,3-bis(4-bromophenyl)propane-1,3-dione (M2) was synthesized according to our previous literature [35,36]. NMR spectra were recorded by Bruker Avance 300 spectrometer, Rheinstetten, Germany, using Deuterochloroform (CDCl_3_) as solvent. Mass spectrometry was collected from a Shimadzu LCMS-2020 Instrument, Kyoto, Japan. Fluorescence spectra were measured on an RF-5301PC spectrometer, Shimadzu, Kyoto, Japan. UV–Vis absorption spectra were undertaken on a Shimadzu UV-3600 spectrophotometer, Kyoto, Japan. Molecular weight of PDBDBM was carried out in THF by gel permeation chromatography (GPC) with a Waters 244 HPLC pump, Milford, MA, USA, THF was served as a solvent and measurements were relative to polystyrene standards. Ultrapure water used in experiments was produced by using a Milli-Q water purification system (Millipore Corp., Billerica, MA, USA).

### 2.2. Preparation of the Conjugated Polymer PDBDBM

M1 (76.6 mg, 0.2 mmol), M2 (77.0 mg, 0.2 mmol), tetrakis(triphenylphosphine)palladium (22.65 mg, 0.02 mmol), and cuprous iodide (3.75 mg, 0.02 mmol) was dissolved in 10 mL dimethylformamide (DMF) and 5 mL Et_3_N. The reaction was performed at 75 °C for two days under N_2_ atmosphere. The solution was filtered through a short silica gel column after the solution cooled to room temperature. Then the solvent was discarded and the precipitates formed were dissolved in 4 mL dichloromethane, and then reprecipitation polymer PDBDBM by adding 60 mL methanol. Finally, the resulting product was precipitated from dichloromethane, filtered, and washed with methanol several times, and dried in a vacuum to give yellow solids in 70% yield.

^1^H NMR (400 MHz, CDCl_3_) δ 7.98 (t, *J* = 7.3 Hz, 2H), 7.87 (d, *J* = 8.2 Hz, 1H), 7.64 (m, 3H), 7.04 (m, 1H), 6.80 (m, 1H), 4.06 (s, 2H), 1.88 (d, *J* = 6.2 Hz, 2H), 1.57 (d, *J* = 7.1 Hz, 2H), 1.30 (m, 9H), 0.88 (m, 3H).

### 2.3. Metal Ion Titration Procedure

Stock of 1 mg/mL PDBDBM was prepared in THF solution and stored at 4 °C until used. Metal ions were dissolved in Milli-Q water for obtaining each metal ion stock solutions (1 × 10^−2^ mol/L). For the metal ion titration experiment, 30 μL PDBDBM (1 mg/mL) was added into 2970 μL THF and thoroughly mixed with the micropipettor. Then different metal ions and different concentrations of Fe^3+^ and Cu^2+^ ions were added to a THF solution of PDBDBM and mixed by pipetting. Finally, the UV–Vis absorption and photoluminescence spectra were recorded. For further probing the structure changes of PDBDBM upon addition of Fe^3+^ and Cu^2+^, NMR spectrum were taken by adding various amounts of Cu^2+^/Fe^3+^ (dissolved in d_6_-DMSO) into a 10 mg/mL solution of PDBDBM in CDCl_3_ solvent.

## 3. Results and Discussion

### 3.1. Synthesis and Characterization of PDBDBM

**Scheme 1:**
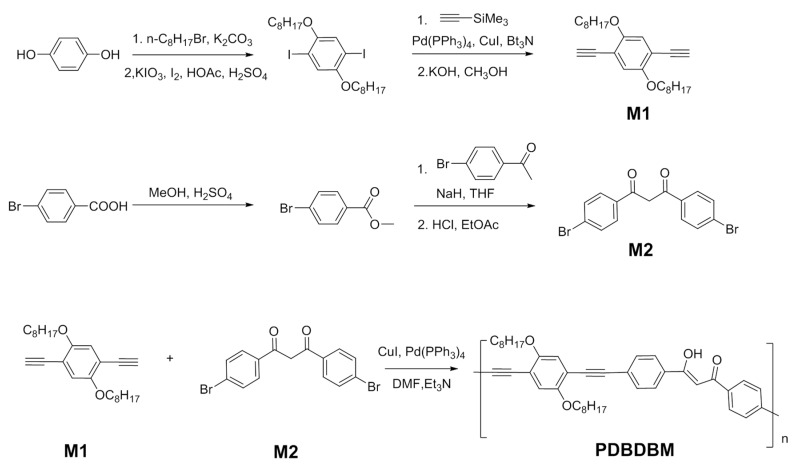
Synthetic routes of PDBDBM.

The synthesis procedure of the monomers and conjugated polymer (PDBDBM) is outlined in Scheme 1. 1,4-dioctyloxy-2,5-diethynylbenzene (M1) was developed from the starting material hydroquinone by a four-step reaction as previously described [35]. 1,3-bis(4-bromophenyl)propane-1,3-dione (M2) was obtained through a three-step reaction from the starting material 4-bromobenzene carboxylic acid as our previously reported literatures [36]. PDBDBM was synthesized from M1 and M2 by Pd-catalyzed Sonogashira-coupling reaction under a mild reaction condition in DMF/Et_3_N (2:1, *v*/*v*) supplement with tetrakis(triphenylphosphine)palladium and cuprous iodide catalysts. To further purify the conjugated polymer PDBDBM, the product was washed with methanol and collected as a solid power in 70% yield. The chemical structure of PDBDBM was confirmed by ^1^H NMR (Figure S1). GPC result showed that the weight-average molecular weight (*M_w_*), the number-average molecular weight (*M_n_*), and polydispersity index (PDI) of PDBDBM are 5657, 2399, and 2.358, respectively (Figure S2). The resulting conjugated polymer exhibits good solubility in common organic solvents—such as DMF, THF, chloroform, and toluene—which is ascribe to the flexible *n*-octyl substituents. UV–Vis absorption and fluorescence spectroscopy of PDBDBM were recorded in THF solution. As shown in Figure S3, PDBDBM has a maximal absorption peak at 420 nm due to the π–π* transition in conjugated polymers; meanwhile, we also find an emission peak at 503 nm from fluorescence spectra.

### 3.2. Selectivity and Sensitivity of the PDBDBM Sensor for Detection of Cu^2+^ and Fe^3+^

**Figure 1:**
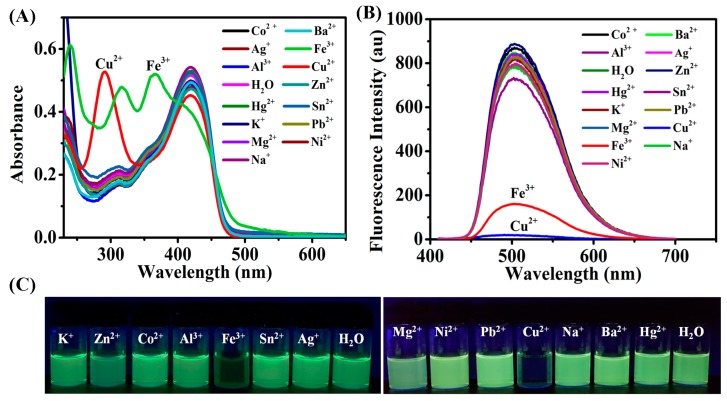
(**A**) UV–Vis spectra of PDBDBM sensor (1 × 10^−5^ mg/mL) and (**B**) fluorescence spectra collected in THF solution after adding various metal ions (150 μM); (**C**) Fluorescence images of PDBDBM sensor (1 × 10^−5^ mg/mL) was taken under UV lamp irradiation after adding various metal ions.

**Figure 2:**
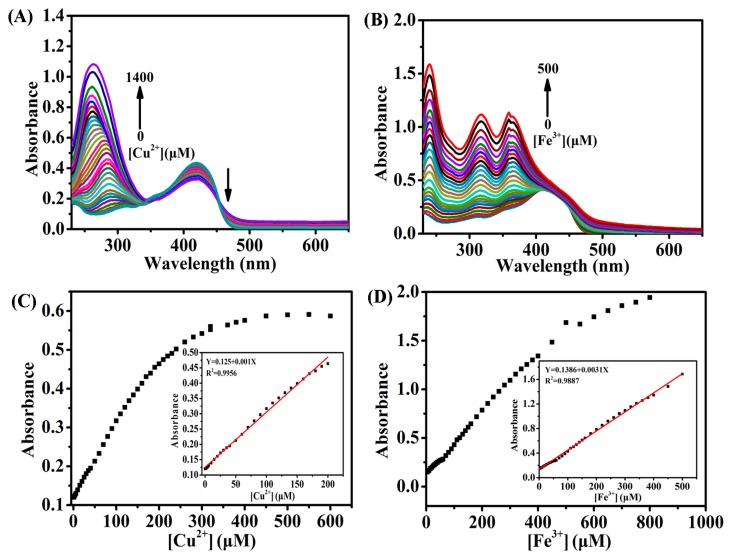
(**A**) UV–Vis absorption responses of PDBDBM sensor (1 × 10^−5^ g/mL) in THF upon addition of Cu^2+^; (**B**) UV–Vis absorption responses of PDBDBM sensor (1 × 10^−5^ g/mL) in THF upon addition of Fe^3+^; (**C**) Absorbance at 288 nm of PDBDBM sensor (1 × 10^−5^ g/mL) in THF versus the increasing concentration of Cu^2+^, the insert shows the plot of absorbance versus the concentration of Cu^2+^; (**D**) Absorbance at 240 nm of PDBDBM sensor (1 × 10^−5^ g/mL) in THF versus the increasing concentrations of Fe^3+^, the insert shows the plot of absorbance versus the concentration of Fe^3+^.

The selectivity and sensitivity of the PDBDBM sensor against different metal ions were carried out by UV–Vis spectrophotometer and luminescence spectrometer. The UV–Vis spectral changes of the PDBDBM sensor (1 × 10^−5^ g/mL) towards different metal ions were investigated in THF solution. As shown in Figure 1A, UV–Vis absorption spectra of the PDBDBM sensor have no significant change by the addition of Co^2+^, Ba^2+^, Ag^+^, Al^3+^, Zn^2+^, Hg^2+^, Sn^2+^, K^+^, Na^+^, Mg^2+^, Ni^2+^, and Pb^2+^. Upon addition of Cu^2+^, the intensity of absorption peak at 420 nm shows a slight reduction with increase the concentration of Cu^2+^. On the contrary, the intensity of absorption peak 314 nm exhibits a gradual heightening and an obvious blue shift (Figure 2A). A good linear dependence of absorbance intensity at 288 nm versus Cu^2+^ concentration ranging from 1 to 200 μM with *R*^2^ = 0.9956 was got as Figure 2C shown. In addition, two new absorbance peaks arise at 240 and 364 nm upon addition of Fe^3+^ ions (Figure 1A). The intensities of absorption peaks at 240, 314, and 364 nm are gradually enhanced with the increasing concentration of Fe^3+^ (Figure 2B). The absorbance intensity of PDBDBM sensor at 240 nm versus Fe^3+^ concentrations exhibits a linear dependence from 0.2 to 500 μM with *R*^2^ = 0.9987 (Figure 2D). Further, the absorbance values at 314 and 364 nm also show dose-dependence ranging from 0 to 200 μM with *R*^2^ = 0.9993 and 0.9998 upon addition of Fe^3+^ (Figure S4C). As Figure S4 shown, the PDBDBM response to Fe^3+^ at 364 nm was hardly influenced by Cu^2+^ (60 μM). This result confirm that the PDBDBM can selectively detect and quantify Fe^3+^ by UV–Vis spectroscopy even in Cu^2+^ and Fe^3+^ mixture. The UV–Vis detection limit of PDBDBM sensor for Cu^2+^ and Fe^3+^ was calculated to be 1.5 μM and 0.15 μM, respectively. These results illustrate that the PDBDBM sensor interaction with Cu^2+^ and Fe^3+^ though different types of binding [37]. These results also show that PDBDBM can be used as a ratiometric sensor for Cu^2+^ and Fe^3+^ detection.

Next, we also checked the selectivity of PDBDBM sensor though fluorescence spectra. Figure 1B demonstrates that most of the tested metal cations (150 μM) did not markedly change the fluorescence intensity, but Cu^2+^ and Fe^3+^ can cause fluorescence quenching. Corresponding fluorescent images were recorded under UV lamp irradiation as Figure 1C shown. Cu^2+^ and Fe^3+^ at 150 μM lead to about 97.7% and 80.8% fluorescence quenching of PDBDBM sensor. The quenching efficiency of Cu^2+^ and Fe^3+^ was 73 and 5.2-fold higher than other metal ions (Figure S5). In addition, we also evaluated the performance of PDBDBM toward to Cu^2+^/Fe^3+^ in metal ions complex solution. As Figure S6 shown, the fluorescence responses of PDBDBM toward to Cu^2+^/Fe^3+^ had no obvious change in complex solution; especially for Cu^2+^. As is well known, a short response time is required for the sensor in most applications [38]. As Figure S7 shown, PDBDBM exhibited a fast response time of 5 s. Therefore, for complete reaction, 10 s of reaction time is selected in this work. These results further confirm that PDBDBM is a potential dual-response and highly selective probe for Cu^2+^ and Fe^3+^ over other metal ions.

**Figure 3:**
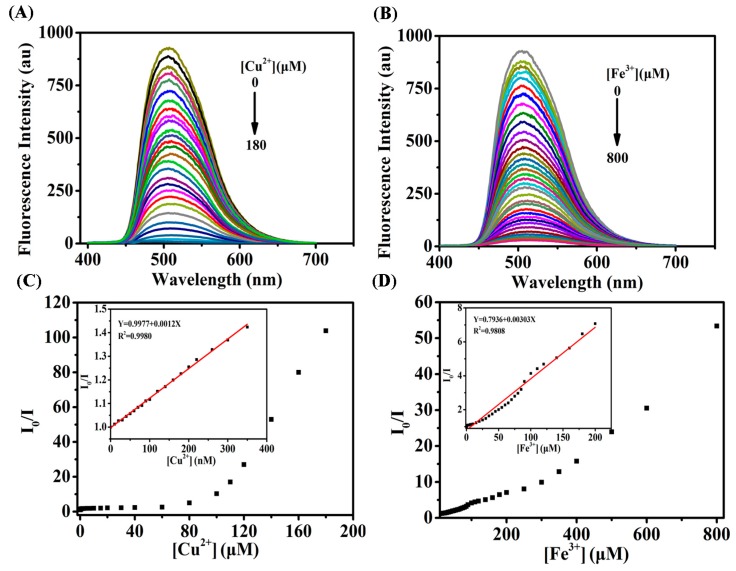
(**A**) Photoluminescence responses of PDBDBM sensor upon addition of Cu^2+^; (**B**) Photoluminescence responses of PDBDBM sensor upon addition of Fe^3+^; (**C**) Photoluminescence quench values (*I*_0_/*I*) versus Cu^2+^ concentration, the Stern–Volmer plots for Cu^2+^ are shown as an inset. (**D**) Photoluminescence quench values (*I*_0_/*I*) versus Fe^3+^ concentration, the Stern–Volmer plots for Fe^3+^ are shown as an inset.

Figure 3A,B depicts the fluorescent responses of PDBDBM sensor upon titration with Cu^2+^ and Fe^3+^. A gradual quenching process in fluorescence intensity of PDBDBM sensor was observed upon progressive addition of Cu^2+^ and Fe^3+^. Over the entire titration process, the Stern–Volmer plots for Cu^2+^ is linear in the concentration range of 10 to 250 nM (Figure 3C); meanwhile, fluorescent quenching value of Fe^3+^ was determined and presents a good linear relationship in the range of 0.6 to 500 μM (Figure 3D). The fluorescence detection limit of PDBDBM sensor for Cu^2+^ and Fe^3+^ was calculated to be 5 nM and 0.4 μM, which are lower than the allowable contaminant level of Cu^2+^ (20 μM) and Fe^3+^ (5.4 μM) in drinking water formulated by the Environmental Protection Agency (EPA) of the USA government [39]. This result indicates that fluorescence response is more sensitive toward small changes that affect electronic properties of molecular receptors [40]. The comparison of our work with the reported chemosensors based on polymers and small organic molecules is shown in Table 1, the present sensor shows high sensitivity for Cu^2+^ and Fe^3+^, especially for Cu^2+^.

As well known, *d*-block ions often create excited state deexcitation pathways via photoinduced electron transfer or electronic energy transfer involving the metal center. Therefore, there are many more examples of turn-off sensors than turn-on [41]. In the present work, the PDBDBM contains an electron accepting acceptor segment (benzene ring) and an electron donor segment (hydroxyl), making acceptor and donor in π conjugation. The PDBDBM sensor shows bright green fluorescence due to the *n*→π* charge transition in the polymer [42]. Besides, the paramagnetic ions with unfilled *d* shells can quench via an electron or energy transfer [15,42]. After PDBDBM–Cu^2+^/Fe^3+^ complex formation, the interaction between PDBDBM and Cu^2+^/Fe^3+^ hinders the *n*→π* charge transition and causes fluorescence quenching [43]. Due to the ligand-to-metal charge transfer, the absorption intensities of PDBDBM at 314 nm increase and blue shift with increased Cu^2+^ concentration and two new absorbance peaks arise at 240 and 364 nm for Fe^3+^ complex (Figure 2). As Figure 2 and Figure 3 show, moreover, there is no overlap between the emission spectrum and the absorption spectrum of the PDBDBM–Cu^2+^/Fe^3+^ complex, further excluding the possibility of fluorescence resonance energy transfer. Hence, the fluorescence quenching may be mainly caused by charge transfer between the PDBDBM and the chelated Cu^2+^/Fe^3+^ [44].

In order to quantify the Cu^2+^ and Fe^3+^ induced quenching process, the Stern–Volmer quenching constant *K_sv_* was calculated by the Stern–Volmer equation, *I*_0_/*I* = 1 + *K_sv_*[Q]. *I*_0_ and *I* are the fluorescence intensities of PDBDBM sensor at maximal emission peak (503 nm) in THF solution with and without quencher, respectively. *K_sv_* is the Stern–Volmer quenching constant, and the concentration of quencher is [Q]. The *K_sv_* value of PDBDBM sensor for Cu^2+^ and Fe^3+^ is 1.28 × 10^8^ M^−1^ and 2.40 × 10^4^ M^−1^, respectively. The *K_sv_* value of PDBDBM for Cu^2+^ is higher than that of poly(pentiptycene) derivatives (2.27 × 10^6^ M^−1^) [45] and poly(fluorine) derivatives (1.44 × 10^7^ M^−1^) [20], and the *K_sv_* value of PDBDBM for Fe^3+^ is about similar to that of julolidine derivatives (3.3 × 10^4^ M^−1^) [46] and rhodamine derivatives (2.46 × 10^4^ M^−1^) [47].

To ascertain the binding mechanism, ^1^H NMR analysis was carried out before and after the formation of PDBDBM-Cu^2+^/Fe^3+^ complexes. As Figure S8 shown, the proton of hydroxy of PDBDBM in absence of Cu^2+^/Fe^3+^ exhibited a sharp signal in ^1^H NMR at 1.88 ppm. The proton signals of hydroxyl became weaker with the increasing concentration of Cu^2+^/Fe^3+^. This phenomenon demonstrates that the oxygen on PDBDBM was directly involved in coordinating with Cu^2+^/Fe^3+^ [42,48].

## 4. Conclusions

In summary, a new polymer-based fluorescent chemosensor probe bearing 1,4-dioctyloxy-2,5-diethynylbenzene and 1,3-bis(4-bromophenyl)propane-1,3-dione was designed and synthesized via Pd-catalyzed Sonogashira-coupling reaction. The conjugated polymer (PDBDBM) shows a high sensitivity and selectivity toward Cu^2+^/Fe^3+^. Upon addition of Cu^2+^ and Fe^3+^, the UV–Vis absorption and PL spectra of PDBDBM could be gradually changed with the increasing concentration of Cu^2+^ and Fe^3+^. The fluorescent detection limit of PDBDBM for Cu^2+^ and Fe^3+^ was measured to be 5 nM and 0.4 μΜ, respectively. The ^1^H NMR result indicates that Cu^2+^/Fe^3+^ ions coordinate with the oxygen of PDBDBM, which is critical for the ligand-to-metal charge transfer. These results further confirm that the conjugated polymer PDBDBM can be used as a potential highly sensitive and selective chemosensor for Cu^2+^/Fe^3+^ detection.

## Figures and Tables

**Table 1:** Comparison of PDBDBM with recently reported chemosensors.

Chemosensors	Target	Detection Type	Limit of Detection	Reference
PDBDBM	Cu^2+^	Fluorescence	5 × 10^−9^ M	This work
Poly(pentiptycene) derivatives	Cu^2+^	Fluorescence	1.65 × 10^−^^8^ M	[45]
Poly(fluorine) derivatives	Cu^2+^	Fluorescence	3.59 × 10^−^^8^ M	[49]
Poly(fluorine) derivatives	Cu^2+^	Fluorescence	2 × 10^−^^8^ M	[20]
Imidazole derivatives	Cu^2+^	Colorimetric	1 × 10^−^^5^ M	[50]
Rhodamine derivatives	Cu^2+^	Fluorescence	3.65 × 10^−^^8^ M	[51]
PDBDBM	Fe^3+^	Fluorescence	4 × 10^−^^7^ M	This work
Poly(fluorine) derivatives	Fe^3+^	Fluorescence	2.2 × 10^−^^7^ M	[4]
Julolidine derivatives	Fe^3+^	Colorimetric	6.8 × 10^−^^6^ M	[46]
Spirooxazine derivatives	Fe^3+^	Fluorescence	8.8 × 10^−^^7^ M	[52]
Rhodamine derivatives	Fe^3+^	Fluorescence	4 × 10^−^^7^ M	[47]
Quinoline derivatives	Fe^3+^	Fluorescence	1.24 × 10^−^^7^ M	[39]

## Supplementary Materials

Supplementary Materials are available online at www.mdpi.com/2073-4360/9/7/267/s1.
