# Supplementary material for: A New Polymer-Based Fluorescent Chemosensor Incorporating Propane-1,3-Dione and 2,5-Diethynylbenzene Moieties for Detection of Copper(II) and Iron(III)
Source: Polymers (Basel). 2017 Jul 6;9(7):267. doi: 10.3390/polym9070267 (PMC6431840; doi:10.3390/polym9070267)
Supplement: Supplementary file 1 [file polymers-09-00267-s001.pdf]

A new polymer-based fluorescent chemosensor incorporating propane-1,3-dione and 2,5-diethynylbenzene moieties for detection of copper(II) and iron(III)

Dongliang Yang[1], Chunhui Dai[2] Yanling Hu[1], Shuli Liu[1], Lixing Weng[3], Zhimin Luo[1], Yixiang Cheng[2,]* Lianhui Wang[1,]*

1 Key Laboratory for Organic Electronics & Information Displays (KLOEID) and Institute of Advanced Materials (IAM), Nanjing University of Posts & Telecommunications, 9 Wenyuan Road, Nanjing 210046, China; 1011071735@njupt.edu.cn (Y.-D.L.); huyanling124@hotmail.com (H.-Y.L.); liushuli_1990@126.com (L.-S.L.); iamzmluo@njupt.edu.cn (L.-Z.M.);

2 Key Lab of Mesoscopic Chemistry of MOE, School of Chemistry and Chemical Engineering, Nanjing University, Nanjing 210093, China; daichunhui@163.com (D.-H.C.);

3 College of Geography and Biological Information, Nanjing University of Posts and Telecommunications, Nanjing 210046, China; lxweng@njupt.edu.cn (W.-L.X.);

* Correspondence: iamlhwang@njupt.edu.cn (W.-L.H.); yxcheng@nju.edu.cn (C.-Y.X.); Tel.: +025-85866332

Contents:

Figure S1 ^1H NMR spectra of the polymer PDBDBM.

Figure S2 GPC curve of the polymer PDBDBM.

Figure S3 UV–vis absorption and PL spectra of the polymer PDBDBM in THF.

Figure S4. (A) UV-vis absorption responses of PDBDBM sensor (1×10^{-5} g/mL) in THF and Cu^{2+} (60 μM) mixture upon addition of Fe^{3+}; (B) UV-vis absorption responses of PDBDBM sensor (1×10^{-5} g/mL) in THF solution upon addition of Fe^{3+}; (C) The plot of absorbance at 314 and 364 nm of PDBDBM sensor versus the concentration of Fe^{3+}; (D) The absorbance values at 364 nm of PDBDBM in different concentrations Fe^{3+} with or without Cu^{2+} (60 μM).

Figure S5 Fluorescence quenching efficiencies of the polymer PDBDBM in the presence of various transition metal ions.

Figure S6. Fluorescence responses of PDBDBM (1.0×10^{-5} g/mL^{-1}) upon addtion of metal ions mixture. The concentration of Cu^{2+} and Fe^{3+} is 50 μM. Mixture: the mixture of Na^{+}, K^{+}, Mg^{2+}, Ba^{2+}, Zn^{2+}, Sn^{2+}, Co^{2+}, Ni^{2+}, Pb^{2+}, Al^{3+} and Ag^{+} (each metal ion finally concentration is 10 μM).

Figure S7 Fluorescence responses of PDBDBM (1.0×10^{-5} g/mL^{-1}) upon addtion of metal ions mixture.

Figure S8 ^1H NMR (400 MHz) spectrum of compound PDBDBM in $CDCl_3$ with Cu^{2+}/Fe^{3+}.

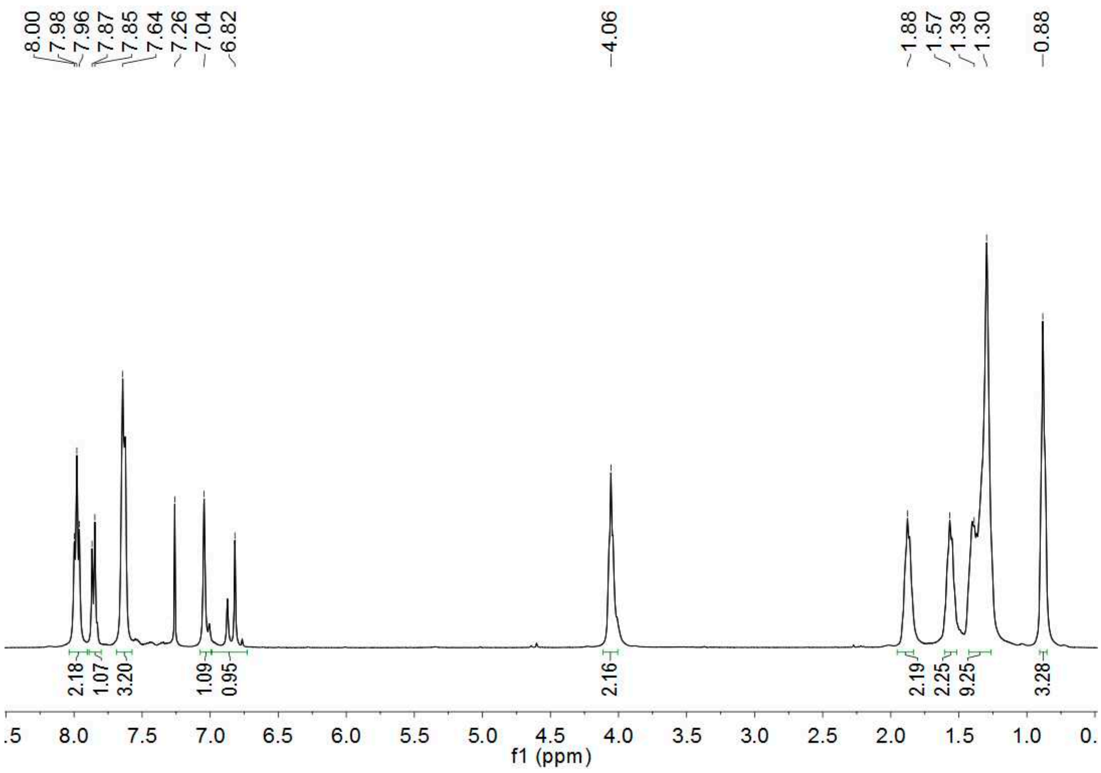

Figure S1 ^{1}H NMR of the polymer PDBDBM ($CDCl_3$, 400 MHz)

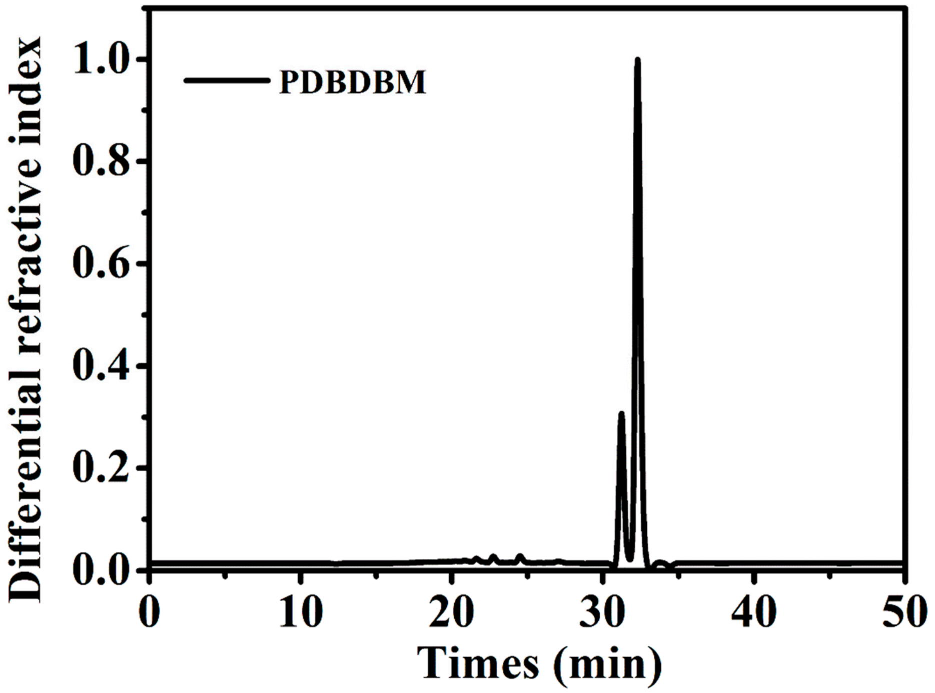

Figure S2 GPC curve of the polymer PDBDBM

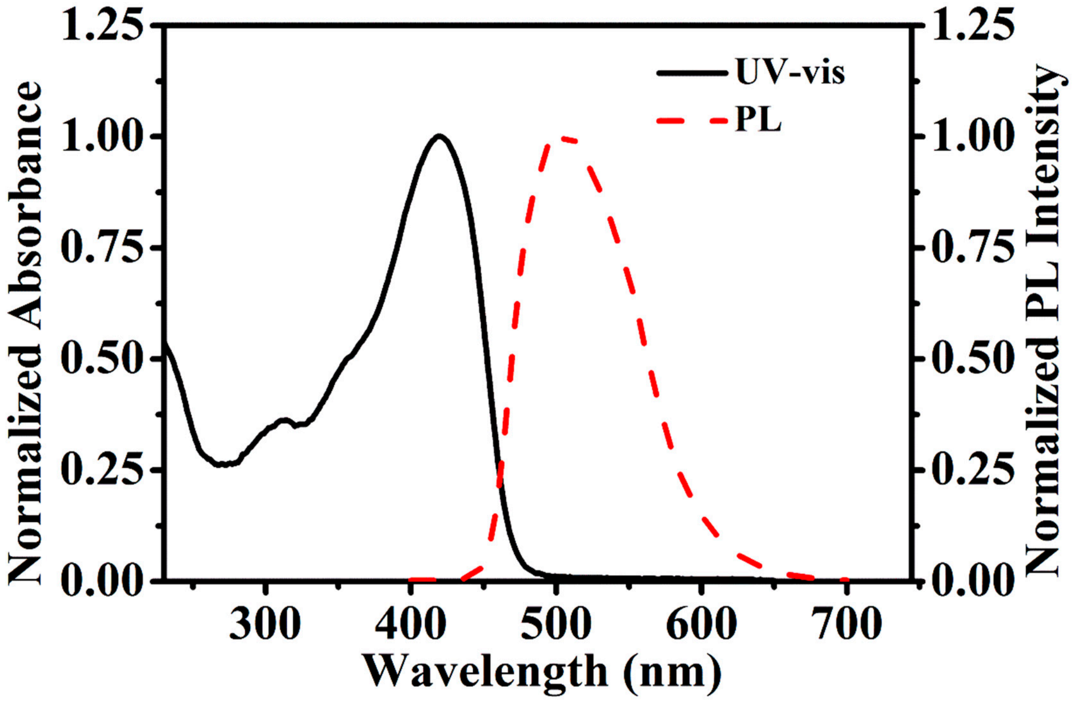

Figure S3. UV–vis absorption and PL spectra of the polymer PDBDBM in THF.

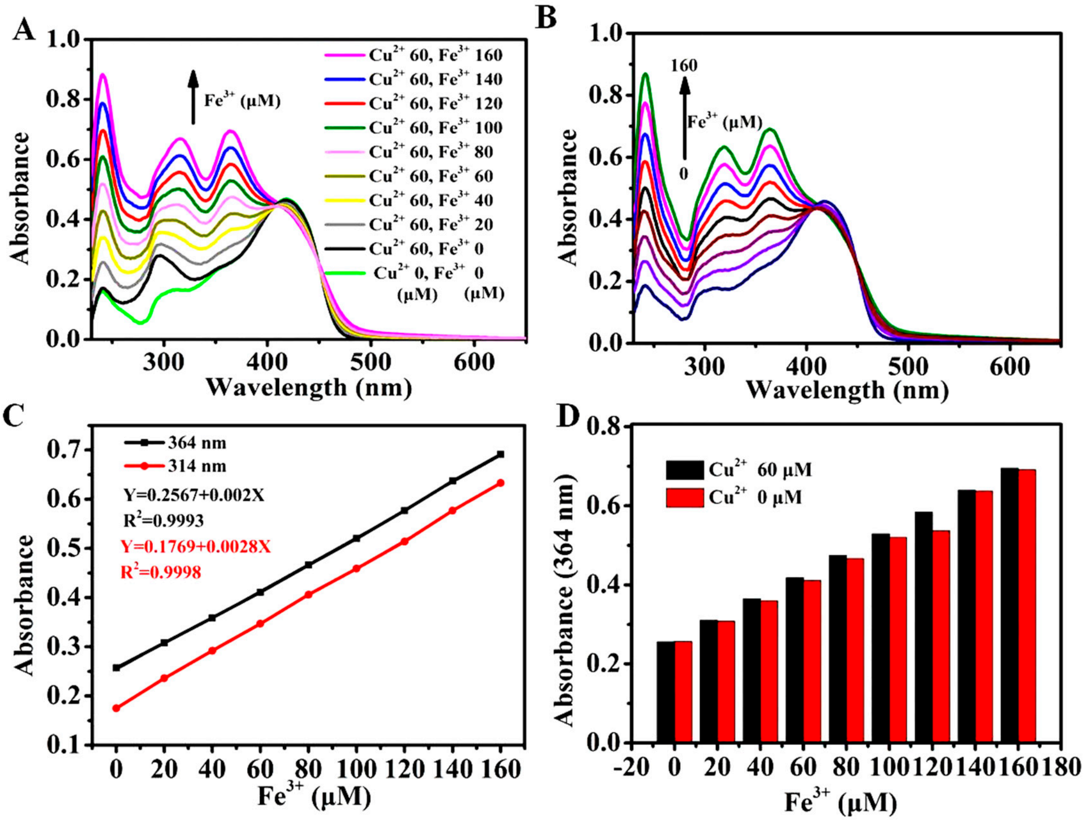

Figure S4. (A) UV-vis absorption responses of PDBDBM sensor (1×10-5 g/mL) in THF and Cu^{2+}

(60 μM) mixture upon addition of Fe^{3+}; (B) UV-vis absorption responses of PDBDBM sensor (1×10^{-5} g/mL) in THF solution upon addition of Fe^{3+}; (C) The plot of absorbance at 314 and 364 nm of PDBDBM sensor versus the concentration of Fe^{3+}; (D) The absorbance values at 364 nm of PDBDBM in different concentrations Fe^{3+} with or without Cu^{2+} (60 μM).

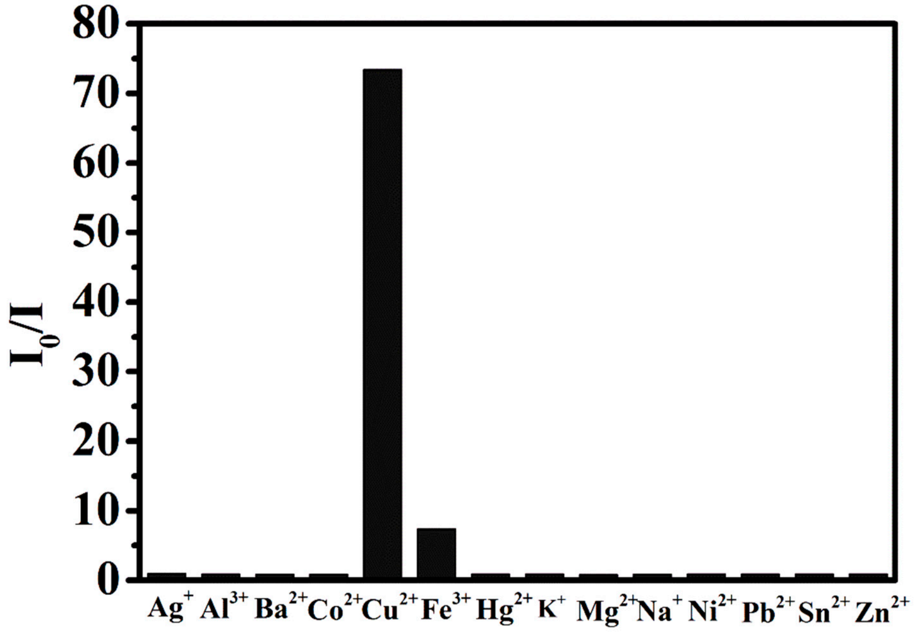

Figure S5. Fluorescence quenching efficiencies of the polymer PDBDBM (1.0×10^{-5} g/mL^{-1}) in the presence of various transition metal ions（150 μM).

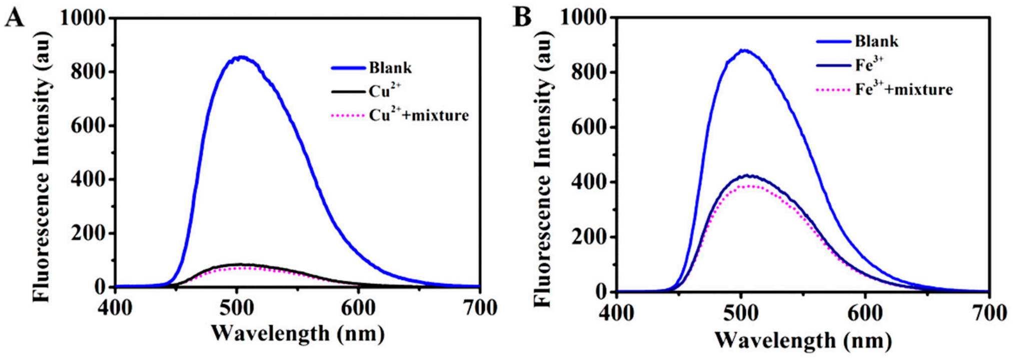

Figure S6. Fluorescence responses of PDBDBM (1.0×10^{-5} g/mL^{-1}) upon addtion of metal ions mixture. The concentration of Cu^{2+} and Fe^{3+} is 50 μM. Mixture: the mixture of Na^+, K^+, Mg^{2+}, Ba^{2+}, Zn^{2+}, Sn^{2+}, Co^{2+}, Ni^{2+}, Pb^{2+}, Al^{3+} and Ag^+ (each metal ion finally concentration is 10 μM)

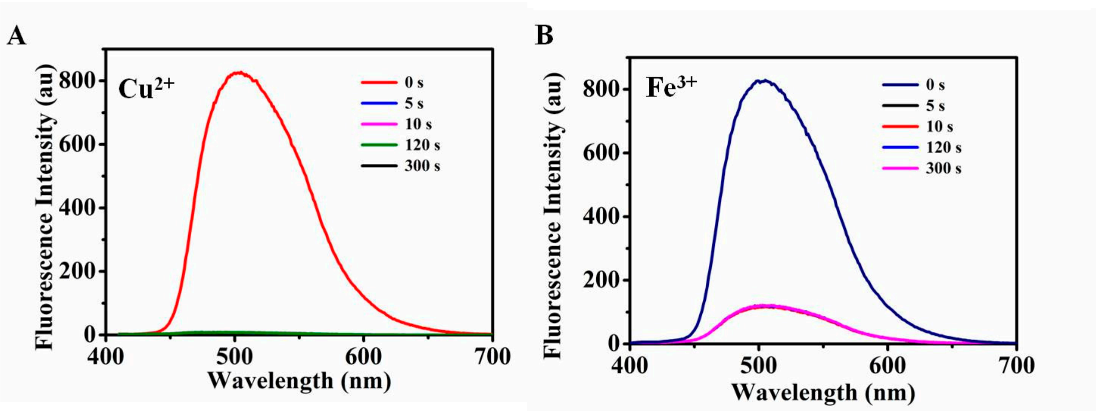

Figure S7. Fluorescence response of PDBDBM (1.0×10^{-5} g/mL^{-1}) to different incubation time upon addition of Cu^{2+}/Fe^{3+} (200 μM).

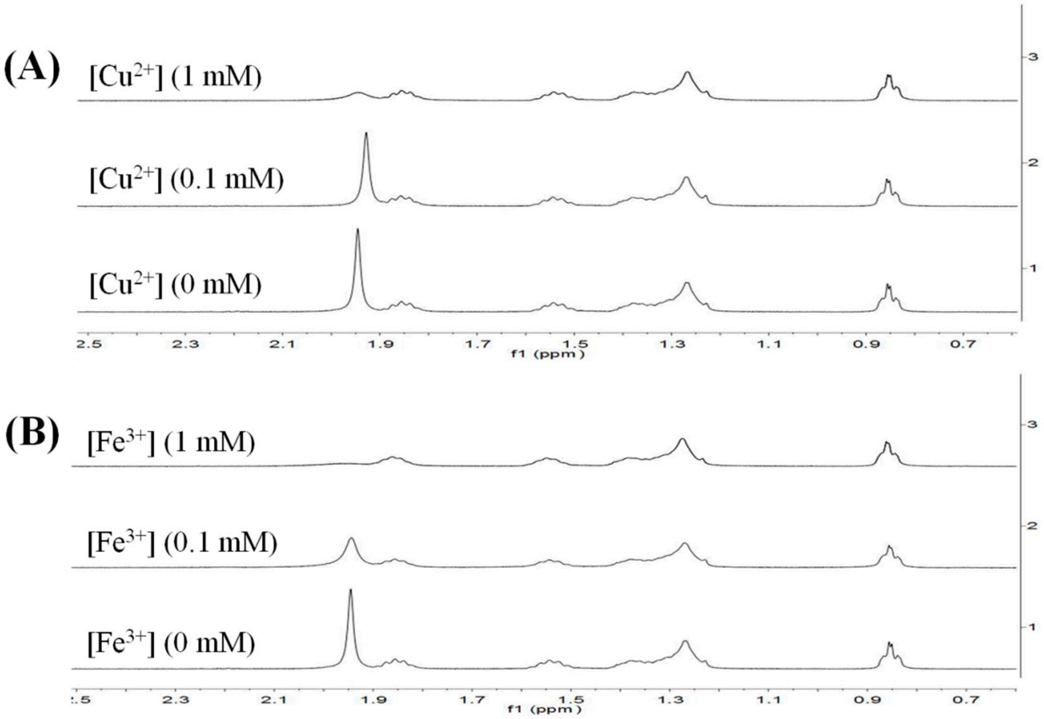

Figure S8. ^{1}H NMR (400 MHz) spectrum of compound PDBDBM in $CDCl_3$ with Cu^{2+}/Fe^{3+} (0, 0.1mM, 1mM, d^6-DMSO).
